# Circular RNA UBE2Q2 promotes malignant progression of gastric cancer by regulating signal transducer and activator of transcription 3-mediated autophagy and glycolysis

**DOI:** 10.1038/s41419-021-04216-3

**Published:** 2021-10-05

**Authors:** Jing Yang, Xing Zhang, Jiacheng Cao, Penghui Xu, Zetian Chen, Sen Wang, Bowen Li, Lu Zhang, Li Xie, Lang Fang, Zekuan Xu

**Affiliations:** 1 Department of General Surgery, The First Affiliated Hospital of Nanjing Medical University, Nanjing, 210029 Jiangsu Province China; 2 Collaborative Innovation Center for Cancer Personalized Medicine, Nanjing Medical University, Nanjing, Jiangsu Province China; 3 Jiangsu Key Lab of Cancer Biomarkers, Prevention and Treatment, Collaborative Innovation Center for Personalized Cancer Medicine, Nanjing Medical University, Nanjing, Jiangsu Province China

**Keywords:** Gastric cancer, Gastric cancer

## Abstract

Gastric cancer remains the third leading cause of cancer-related mortality worldwide. Emerging evidence has shown that circular RNAs (circRNAs) play a critical regulatory role in the occurrence and development of various cancers through sponging miRNAs or acting as RNA-binding protein (RBP) sponges. We found that circUBE2Q2 was significantly upregulated in GC tissues and cell lines. Knockdown of circUBE2Q2 inhibited proliferation, migration, invasion, and glycolysis, and increased autophagy in vitro. In addition, knockdown of circUBE2Q2 inhibited GC tumorigenicity and metastasis potential in vivo. A series of experiments were performed to confirm that circUBE2Q2 regulates GC progression via the circUBE2Q2-miR-370-3p-*STAT3* axis and promotes tumor metastasis through exosomal communication. Further in vivo experiments confirmed that, combination treatment of circUBE2Q2 knocking down and *STAT3* inhibitor has synergistic effects on the gastric cancer growth inhibition, which provides a possibility to enhance the sensitivity of targeted drugs to gastric cancer through targeting circUBE2Q2. Our findings revealed that circUBE2Q2 may serve as a new proliferation-promoting factor and prognostic marker in gastric cancer.

## Introduction

Gastric cancer (GC) is the fifth most common cancer and malignant tumor, which ranks as the third leading cause of cancer-related mortality worldwide according to the GLOBOCAN database [1]. In the past few years, although many advances have been achieved in terms of diagnostic approaches and surgical procedures, the high recurrence rate and poor 5-year survival rate of GC remain unsatisfactory [2–4]. Hence, investigation of the underlying molecular mechanisms of the malignant progression of GC is of great concern for improving the survival of patients and reducing the recurrence rate.

Circular RNAs (circRNAs), which are derived from back-splicing of precursor mRNA transcripts, are a novel class of noncoding endogenous RNAs that have a unique single-stranded closed loop and lack both 5′–3′ polarity and a polyadenylated tail [5, 6]. Owing to the development of next-generation sequencing and bioinformatic methods, more functional circRNAs have been identified [7]. An increasing number of studies have shown that circRNAs participate in many biological processes, such as proliferation, apoptosis, and migration, and participate in the initiation and development of various cancers [8–10]. MicroRNAs (miRNAs) are also a class of noncoding single-stranded RNA molecules with a length of about 22 nucleotides encoded by endogenous genes, which are involved in the regulation of posttranscriptional gene expression [11]. Emerging evidence suggests that circRNA can function as miRNAs sponges to regulate downstream genes in tumors [5, 12]. Furthermore, it has been reported that some circRNAs can act as RNA-binding protein (RBP) sponges or function as protein translation [12–14]. However, the potential mechanism of circRNA regulating the malignant behavior of GC is still unclear and deserves further investigation.

The signal transducer and activator of transcription 3 (*STAT3*) is considered as a bona fide oncogene that belongs to the STAT protein family [15]. A growing body of evidence has implicated that aberrant activation of *STAT3* participates in tumorigenesis in terms of regulating autophagy, cell cycle, glycolysis, and metastasis [16–19]. *STAT3* may upregulate *Bcl-2* expression to inhibit autophagy, further promoting the proliferation of cancer cells [19–22]. Moreover, activation of *STAT3* is associated with advanced cancer stage and metastatic disease [23–26].

Emerging evidence has identified that exosomes are involved in mediating material transfer and molecular communication between primary tumor sites and distant metastasis sites [27]. Many circRNAs have been reported to promote or suppress tumors via exosomes [28, 29].

In our study, we proved that circUBE2Q2 is upregulated in GC tissues and cell lines, sponges miR-370-3p to activate the *STAT3* pathway, and promotes tumor metastasis through exosomal communication, eventually promoting the malignant progression of GC.

## Materials and methods

### Tissue samples

All GC tissues and adjacent normal tissues were collected from GC patients who underwent surgery in the Department of Gastric Surgery, the First Affiliated Hospital of Nanjing Medical University from 2013 to 2018. None of the patients received preoperative chemotherapy or radiotherapy. All samples were frozen in liquid nitrogen immediately and stored at −80 °C until use; diagnoses were validated through pathological analysis. Clinicopathological features, including gender, age, T stage, lymphatic invasion, tumor site, size, stage, and histology grade, are shown in Table 1. This study was approved by the Clinical Research Ethics Committee of the First Affiliated Hospital of Nanjing Medical University.

**Table 1:** Clinicopathological features of 60 gastric cancer patients and the expression of circUBE2Q2.

Parameters	Group	circUBE2Q2 expression			
		Cases	Low	High	*P*-value
Gender	Female	20	8	12	0.2733
	Male	40	22	18	
Age at surgery (years)	≥50	48	23	25	0.5186
	<50	12	7	5	
T grade	T1 + T2	18	6	12	0.091
	T3 + T4	42	24	18	
Lymphatic invasion	Negative(N0)	10	8	2	0.0377*
	Positive (N1–N3)	50	22	28	
Tumor site	Cardiac	27	14	13	0.7952
	Non-cardiac	33	16	17	
Stage	I–II	21	12	9	0.4168
	III–IV	39	18	21	
Size (cm)	<3	35	22	13	0.0184*
	≥3	25	8	17	
Histology grade	Intestinal type	29	13	16	0.4383
	Diffuse type	31	17	14	

*P < 0.05 represents statistical significance (*χ*^2^-test).

### RNA sequence

Total RNA was extracted from three pairs of frozen GC tissues and paired normal tissues using TRIzol Reagent (Invitrogen, CA, USA). Ribosomal RNAs were removed using a RiboMinus Eukaryote kit (Qiagen, Valencia, CA). Then RNA was treated with RNase R (Epicentre, USA) and fragmented to ~200 bp. cDNA was synthesized with random hexamer primers and dUTPs. Uracil DNA glycosylase was used to purify cDNA. Next, the RNA sequencing (RNA-seq) library was deep sequenced with an Illumina HiSeq 3000 instrument (Illumina, San Diego, CA).

### Cell culture procedures

The human GC cell lines BGC-823, SGC-7901, MKN-45, MGC-803, HGC-27, and normal GES-1 stomach mucosa epithelium cell lines were cultured in RPMI-1640 (Wisent, Shanghai, China) supplemented with 10% fetal bovine serum (FBS) and 1% penicillin–streptomycin. AGS was cultured in F-12K (Wisent, Shanghai, China) supplemented with 10% FBS and 1% penicillin–streptomycin. These cell lines were incubated at 37 °C in a humidified atmosphere with 5% CO_2_.

### RNA isolation, reverse transcription, and qRT-PCR

Total RNA from paired tissues was extracted by using TRIzol reagent (Invitrogen). We reverse-transcribed RNA into cDNA using PrimeScript RT Reagent (TaKaRa, RR036A, Japan). The calculated Ct values were normalized against those of β-actin to confirm the accurate expression of the circRNA. The −ΔCt method was used to estimate the difference value (ΔCt = Ct tested − Ct β-actin). We analyzed each sample and repeated all of the reactions three independent times, to ensure that all of the data were repeatable. β-Actin and U6 were used as an internal control for RNA. The PCR primer sequences were synthesized by Realgene (Nanjing, China) and Ribobio (Guangzhou, China), which are listed in Supplementary Table 1.

### RNase R treatment

RNase R digestion was carried out at 37 °C for 20 min by incubating the total RNA with 3 U/mg RNase R (Epicentre Technologies, Madison, WI, USA) to remove the linear RNA. The expression of circUBE2Q2 and UBE2Q2 was detected via quantitative real-time PCR (qRT-PCR).

### Actinomycin D assay

BGC-823 and MKN-45 cells were treated with 3 mg/L actinomycin D (Act D; Sigma-Aldrich, St. Louis, MO, USA) and total RNA was collected at the indicated time points (0, 4, 8, 12, and 24 h). Then, the stability of circUBE2Q2 was detected via qRT-PCR.

### Isolation of nuclear and cytoplasmic fractions

The nuclear and cytoplasmic fractions were extracted using NE-PER Nuclear and Cytoplasmic Extraction Reagents (Thermo Fisher Scientific, USA) according to the manufacturer’s protocol.

### Oligonucleotide transfection

MKN-45 and BGC-823 cells were transfected with Lipofectamine 3000 (Thermo Fisher Scientific, Waltham, MA, USA) at a final concentration of 50 nM. After transfection for 48 h, cells were collected for further investigation. Small interfering RNA (siRNA), miRNA inhibitors, and mimics were synthesized by RiboBio (Guangzhou, China). The detailed sequences used are listed in Supplementary Table 1. To construct overexpression plasmids for circUBE2Q2, cDNA was synthesized and cloned into the pcDNA3.1 vector (RiboBio, Guangzhou, China).

### Cell counting kit-8

In 96-well plates, 10,000 specific MKN-45 and BGC-823 cells were inoculated per well. After that, we added 10 μl of Cell counting kit-8 (CCK-8) solution (Dojindo Laboratories, Kumamoto, Japan) to each well within a specified time (24, 48, 72 h, etc.) according to the manufacturer’s protocol. After 2 h of incubation, the absorbance at 450 nm was measured with a microplate reader (BioTek, Winooski, VT, USA).

### Colony formation assay

MKN-45 and BGC-823 cells were separately digested into cell suspensions by trypsin and inoculated in different six-well plates. After 2 weeks of culture, these cells were fixed with 4% paraformaldehyde (PFA) and then stained with crystal violet before being observed.

### Transwell assay

In the cell migration assay, Transwell compartments (Millipore, Billerica, MA, USA) without Matrigel were placed in a 24-well plate. Then, we added 200 μl serum-free RPMI-1640 to the upper compartments and 600 μl complete medium to the lower compartments. After incubation for 24 h at 37 °C, the cells were fixed with 4% PFA and stained with crystal violet. The method of measuring cell invasion is the same with the addition of Matrigel (BD Biosciences) to the upper compartments.

### 5-Ethynyl-2’-deoxyuridine assay

We seeded cells in the logarithmic growth phase in a 96-well plate at 10,000 per well and cultured them under normal conditions. Then, a certain amount of 5-ethynyl-2’-deoxyuridine (EdU) solution (RiboBio, China) was added to each well for 2 h and 4% PFA was used to fix the cells after EdU labeling. After that, we added glycine, penetrant (0.5% Triton X-100), and Apollo reaction solution to each well. Finally, we stained the cells with Hoechst 33342 and observed them under a fluorescent microscope.

### Dual-luciferase reporter assay

Luciferase reporter vectors (pmirGLO) containing circUBE2Q2-miR-370-3p binding sequences or mutant sequences and miRNA mimics were transfected into GC cells using Lipofectamine 3000, to examine their miRNA-binding ability. After 48 h of incubation, the luciferase activity was measured using a dual-luciferase reporter assay system kit (Promega, USA) according to the manufacturer’s protocol. Each experiment was repeated at least three times.

### Mouse xenograft model

Four-week-old female nude mice were purchased from the Laboratory Animal Center of Nanjing Medical University. Then, the nude mice were inoculated subcutaneously in the inguinal region with 1 × 10^6^ stably transfected GC cells (MKN-45 sh-NC, MKN-45 sh-circUBE2Q2, BGC-823 sh-NC, and BGC-823 sh-circUBE2Q2) suspended in 0.1 ml phosphate-buffered saline (PBS). After 1 week, pre-established tumor xenografts were treated with dimethyl sulfoxide or stattic (50 mg/kg, 2×/wk × 3) (MCE, HY-13818, China). Tumor volumes were measured once a week. After 3 weeks, all of the nude mice were killed and then the xenograft tumors were collected to measure their weights. Tumor volume was calculated by 1/2 (length × width [2]). All experiments were approved by the Ethics Committee of Nanjing Medical University.

### Total exosome isolation

All exosome isolation (from cell culture media) reagents (4478359, Invitrogen) were purchased from Invitrogen (USA). We followed the exosome isolation procedures according to the manufacturer’s instructions. First, we centrifuged the cell media at 2000 × *g* for 30 min to remove cells and debris, and transferred the supernatant containing the cell-free culture media to a new tube without disturbing the pellet, which was followed by adding 0.5 volumes of the Total Exosome Isolation (from the cell culture media) reagent. Then, we incubated the samples at 4 °C overnight and centrifuged the samples at 10,000 × *g* for 1 h at 4 °C the next day. Subsequently, we resuspended the samples in a convenient volume of 1 × PBS. After the exosomes were isolated, total RNA and protein were purified by the Total Exosome RNA and Protein Isolation Kit (4478545, Invitrogen).

### Fluorescent in situ hybridization

Cells were fixed with 4% PFA for 15 min at room temperature (RT), washed twice with PBS and incubated in 70% ethanol overnight at 4 °C. Then, the cells were permeabilized with 0.25% Triton X-100 for 15 min and washed with selenium citrate (SSC) buffer twice (15 min each). Cells were handled overnight at 37 °C with biotin-labeled DNA oligo probes in hybridization buffer and washed with SSC buffer several times. Next, samples were incubated in blocking buffer (1% bovine serum albumin (BSA) and 3% normal goat serum in PBS) for 1 h at RT and incubated with an antibiotin horseradish peroxidase (HRP) antibody (1 : 200) in blocking buffer overnight at 4 °C. Subsequently, the samples were washed with PBS twice and the DNA was stained with 4′,6-diamidino-2-phenylindole (DAPI). Finally, we captured images using a fluorescence microscope (Olympus BX53, Olympus America, Inc., Center Valley, PA, USA).

### Human gastric organoid model

We established a human gastric organoid model to simulate the microenvironment of the human body on the basis of a previous article [30]. We transfected an overexpression plasmid and siRNA targeting circRNA into the organoid model, to investigate its effect on GC. Every 4 days, we replaced the media with fresh nutrient solution. Finally, we used a microscope to observe the growth of the human GC organoid model daily.

### Immunofluorescence assay

Cells were fixed with 4% PFA for 10–15 min at RT. Then, the cells were incubated with blocking solution (0.8× PBS, 0.5% Triton X-100, 50 mM NaCl, 3% BSA) for 1 h. Next, we incubated the cells with the primary antibody in blocking solution overnight at 4 °C. The next day, the cells were washed three times for 10 min in 0.8× PBS, 50 mM NaCl, and 1.5% BSA at RT. Then, the cells were incubated with the secondary antibody goat anti-rabbit IgG (Jackson, 1 : 100) in the dark for 2 h at 37 °C in blocking solution, followed by three washes for 10 min in 0.8× PBS, 0.5% Triton X-100, and 50 mM NaCl. Finally, the cells were washed in PBS, counterstained using DAPI, and stored in the dark at 4 °C.

### Immunohistochemical analysis of tissue samples

We used 10% formalin to fix GC tissues and embedded them in paraffin. Then, we cut the GC tissues into slices and incubated them with the primary antibody overnight at 4 °C. Subsequently, we used PBS to wash them twice and used HRP-polymer-conjugated secondary antibody (Abcam, UK) to incubate the tissues at RT for 1 h. Next, we used 3,3-diaminobenzidine solution and hematoxylin to stain the samples. Last, we used a microscope to observe the samples. Immunohistochemical (IHC) score = intensity × frequency score. The intensity of cell staining was graded as 0 (negative), 1 (weak), 2 (moderate), and 3 (strong), and frequency was graded by the percentage of positive cells, which was grade 0, <5%; grade 1, 5 ~ 25%; grade 2, 25 ~ 50%; grade 3, 50 ~ 75%; grade 4, >75%.

### Transmission electron microscopy

For the exosomes, we loaded purified exosomes from MKN-45 and BGC-823 cells onto firmware carbon-coated grids and fixed them with 2% PFA. We then used 2.5% glutaraldehyde to further fix the exosomes and then embedded them in a mixture of methyl cellulose (0.13%) and uranyl acetate (0.4%).

For the autophagosomes, we fixed MKN-45 and BGC-823 GC cells with 2.5% glutaraldehyde at 4 °C overnight. Subsequently, we used 1% OsO_4_ to fix the cell samples. In the following step, we used ethanol and propylene oxide to dehydrate the cell samples. We then stained them with 0.3% lead citrate. Finally, a JEM-1010 electron microscope (JEOL, Tokyo, Japan, ×2500 or ×8800 magnification) was used to detect exosomes and autophagosomes.

### Protein extraction and western blotting analysis

RIPA lysis solution (20 mM Tris-HCl buffer pH 7.5, containing 1 mM EDTA, 1 mM dithiothreitol, 1 mM phenylmethylsulfonyl fluoride, and 1 mM protease inhibitor cocktail) was used to extract total protein from cells or tissues. Then, we used the Bicinchoninic Acid Assay (BCA) method to determine the protein quantity. The samples were separated using 10% SDS-polyacrylamide gel electrophoresis with a constant voltage of 120 V using an electrophoresis apparatus (Bio-Rad, America). The proteins were transferred to polyvinylidene difluoride membranes. Next, the membranes were incubated with the primary antibodies at 4 °C overnight. Then, the membranes were washed using Tris-HCI Buffer Salt Solution + Tween20 (TBST) three times and incubated with the secondary antibody at RT for 2 h. The details of all antibodies are listed in Supplementary Table 2.

### RNA pull-down assay

We incubated the C-1 magnetic beads (Life Technologies) with the circUBE2Q2 probe at 25 °C for 2 h. GC cells were subsequently sonicated into cell lysates. We incubated the probe-coated beads with cell lysate at 4 °C for one night. We isolated the RNA mix bound to the beads for qRT-PCR.

### Glucose, ATP, and lactate assays

For the glucose uptake assay, we incubated GC cells with 100 μM 2-NBDG (11,046, Cayman) for 30 min and washed them with precooled PBS. We then used FL-1 fluorescence to measure glucose uptake. For the ATP assay and lactate assay, we determined the intracellular ATP in the cell extracts via an ATP assay kit (S0026, Beyotime) and determined the lactate level in the cell lysate via a lactate assay kit (K627, BioVision).

### Extracellular acidification rate measurements

Glycolytic capacity was determined via a Seahorse XF24 analyzer (Seahorse Biosciences).

### In vivo metastasis assay

Four-week-old female nude mice were purchased from the Laboratory Animal Center of Nanjing Medical University. In the nude mice liver or peritoneal metastasis model, 1 × 10^6^ GC cells were injected into BALB/c nude mice through their hepatic portal veins or abdominal cavity, respectively, and then indicated treatment including exosomes containing luciferase-labeled circUBE2Q2 were given to the mice. Subsequently, BALB/c nude mice were injected with 100 mg/kg d-luciferin (Xenogen, Hopkinton, MA) and then they underwent bioluminescent scans via an IVIS 100 Imaging System (Xenogen).

### Statistical analysis

All data are reported as the mean value ± SD. GraphPad Prism (version 8.0) was used for general statistical analysis. Differences between the two groups were analyzed via Student’s *t*-test. The correlation of circUBE2Q2 expression with clinicopathological features was analysed via the *χ*^2^-test. *P* < 0.05 was considered statistically significant.

## Results

### Identification of circUBE2Q2 in GC

**Fig. 1:**
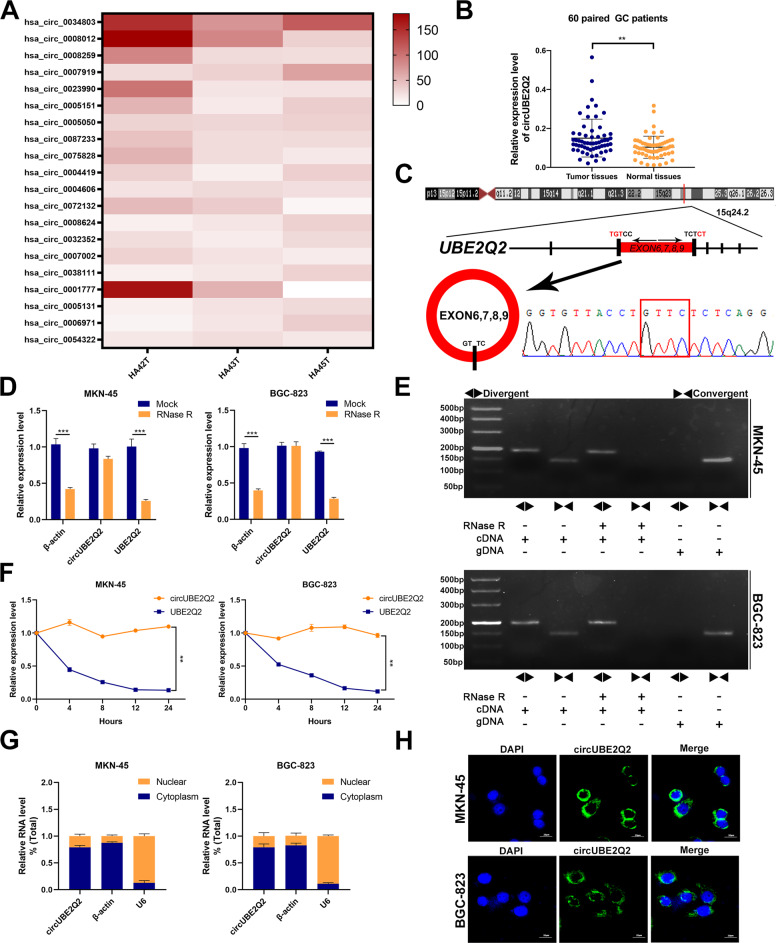
Identification of circUBE2Q2 in GC. **A** Clustered heatmap showing the top 20 differentially expressed circRNAs in three paired human gastric cancer tissues relative to paired normal tissues. **B** qRT-PCR expression of circUBE2Q2 in GC tissues relative to matched normal tissues (*n* = 60). **C** Sanger-sequencing-validated circular structure of circUBE2Q2. The arrows indicate the splicing sites of circUBE2Q2. **D** qRT-PCR quantified the abundance of circUBE2Q2 and linear UBE2Q2 in MKN-45 and BGC-823 cells treated after RNase R treatment. **E** Agarose gel electrophoresis assay for products of linear and back-splicing amplified with convergent and divergent primers with and without RNase R treatment. **F** qRT-PCR analysis of circUBE2Q2 and *UBE2Q2* after treatment with Actinomycin D at the specified time points. **G** qRT-PCR analysis of the cytoplasm and nucleus of circUBE2Q2, respectively. **H** Fluorescence in situ hybridization (FISH) for circUBE2Q2 localization, scale bar = 20 µm. Nuclei were stained blue with DAPI. Data are expressed as the mean ± SD. *p < 0.05, **p < 0.01, ***p < 0.001.

To construct a circRNA profiling database of GC patients, we collected three clinical GC tissues and their paired adjacent normal tissues to perform RNA-seq analyses. We detected 450 differentially expressed circRNAs in the GC tissues and among the 450 differentially expressed circRNAs (fold change ≥ 2 or ≤ 0.5, *P* < 0.05), 314 were upregulated and 136 were downregulated. The top 20 upregulated circRNAs, which are employed in CircBase (http://circrna.org/), are shown via a cluster heatmap (Fig. 1A). We next detected the expression of the top ten circRNAs in GC cell lines (BGC-823, SGC-7901, AGS, MKN-45, MGC-803, HGC-27, and GES-1 cell lines). Circ_0005151 (termed circUBE2Q2 in the remainder of the article), which was significantly upregulated among these ten circRNAs, attracted our attention (Supplementary Fig. 1A, B). To confirm whether the expression level of circUBE2Q2 in GC tissues was consistent with the RNA-seq data, we collected an additional cohort with 60 pairs of GC tissues and adjacent normal tissues, and detected higher expression of circUBE2Q2 in GC tissues via qRT-PCR (Fig. 1B). BGC-823 cells showed the highest expression of circUBE2Q2 and the expression of circUBE2Q2 in MKN-45 cells was the second highest (Supplementary Fig. 1A). Therefore, we selected the BGC-823 and MKN-45 cell lines as the follow-up research objects.

The Sanger-sequencing results showed that circUBE2Q2 is derived from the parental gene *UBE2Q2*, which is located on chromosome 15 and consists of the head-to-tail splicing of exons 6–9 (76168528–76175765) (Fig. 1C). Compared with its parental linear *UBE2Q2* or actin amplified by convergent primers, circUBE2Q2 amplified by divergent primers could resist digestion by RNase R, which indicated that circUBE2Q2 is significantly less susceptible to RNase R digestion than linear *UBE2Q2* (Fig. 1D). Subsequently, an agarose gel electrophoresis assay of the PCR products was performed to detect the expression levels of the back-spliced or canonical forms of *UBE2Q2* with or without RNase R treatment (Fig. 1E). CircUBE2Q2, rather than linear *UBE2Q2*, could be detected in cDNA even under RNase R treatment but not in gDNA, which suggested that circUBE2Q2 was not attributable to genomic rearrangements or PCR artifacts. We confirmed that the genomic size and sequence of circUBE2Q2 in its PCR products were consistent with those in the CircBase database (Fig. 1C). Furthermore, we added Act D, a transcription inhibitor, and found that circUBE2Q2 was more stable than linear *UBE2Q2* (Fig. 1F). qRT-PCR analysis after separation of the nuclear and cytoplasmic RNA and fluorescence in situ hybridization (FISH) analysis confirmed that circUBE2Q2 was predominately located within the cytoplasm of GC cells (Fig. 1G, H). In addition, when we collected the clinical data of the aforementioned patients, the expression level of circUBE2Q2 was found to be significantly correlated with the patients’ tumor size and lymphatic invasion (Table 1). Taken together, these results indicated that circUBE2Q2 is a stable cytoplasmic circRNA and may be a meaningful diagnostic or prognostic marker worthy of further study.

### CircUBE2Q2 promotes the proliferation, migration, and invasion of GC cells in vitro

**Fig. 2:**
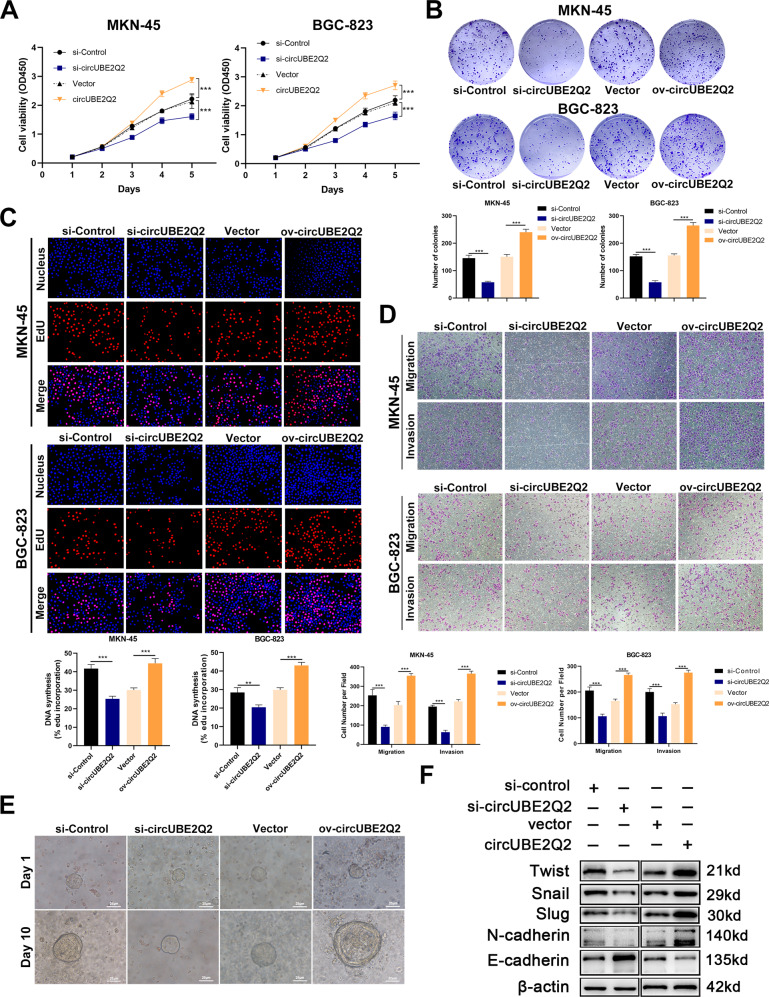
CircUBE2Q2 promotes the proliferation, migration, and invasion of GC cells in vitro. **A** Assessment of the proliferation of MKN-45 and BGC-823 cell lines transfected with circUBE2Q2-specific siRNA or an overexpression plasmid by CCK-8 assay. **B** Assessment of cell proliferation by colony formation assay. **C** Assessment of cell proliferation by EDU assay. **D** Assessment of migration and invasion of by transwell assay. **E** Gastric cancer organoid models indicating the effect of circUBE2Q2 on the growth of GC, scale bar = 25 µm. **F** The protein level of EMT-related genes (TWIST, Snail, Slug, N-cadherin, and E-cadherin) in GC organoid models detected by western blotting. Data are expressed as the mean ± SD. *p < 0.05, **p < 0.01, ***p < 0.001.

To verify whether circUBE2Q2 could affect biological behavior of GC cells, we observed the biological function of circUBE2Q2 by transfecting MKN-45 and BGC-823 cells with circUBE2Q2 siRNA or circUBE2Q2 overexpression plasmids. The transfection efficiency was confirmed by qRT-PCR, meanwhile the mRNA level of *UBE2Q2* (linear *UBE2Q2*) was not influenced (Supplementary Fig. 1C). We observed that overexpression of circUBE2Q2 promoted cell proliferation via CCK-8 assays (Fig. 2A) and also could promote colony formation ability (Fig. 2B) and DNA synthesis, as indicated by the EdU assay (Fig. 2C) in MKN-45 and BGC-823 cells. Furthermore, circUBE2Q2 overexpression promoted the migration and invasion of GC cells via Transwell assays, indicating that circUBE2Q2 was relevant to GC metastasis (Fig. 5D). The opposite effects were observed when we used circUBE2Q2 siRNA in terms of GC cell proliferation and metastasis (Fig. 2A–D). Moreover, we found that circUBE2Q2 could significantly promote the growth of gastric organoids (Fig. 2E). Epithelial to mesenchymal transition (EMT) is closely correlated with GC cell migration and invasion [31, 32]. Thus, we applied western blottings to investigate the role of circUBE2Q2 in the regulation of EMT-related factors. Downregulated mesenchymal cell markers TWIST, snail, slug, and N-cadherin, and upregulated epithelial cell marker E-cadherin were observed in GC organoid models when we knocked down circUBE2Q2 (Fig. 2F).

Taken together, these results validated the tumor promotor role of circUBE2Q2 in GC cells and organoid models via the promotion of proliferation and metastasis.

### CircUBE2Q2 promotes GC development by serving as a miRNA sponge of miR-370-3p

**Fig. 3:**
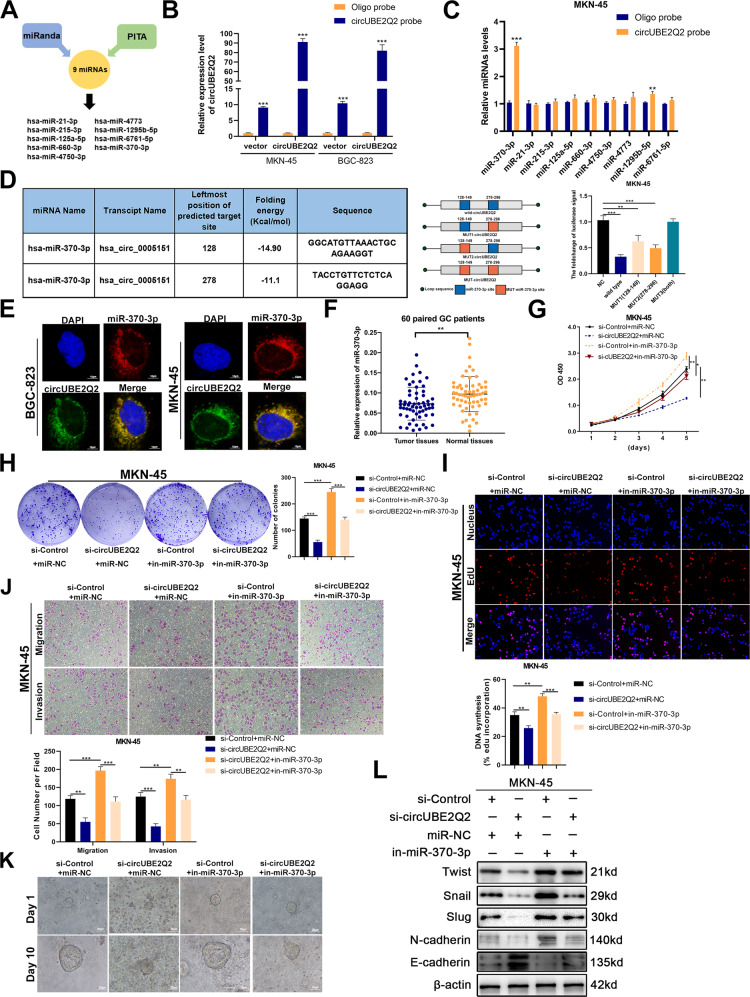
CircUBE2Q2 promotes GC development by serving as a miRNA sponge of miR-370-3p. **A** Venn diagram showing the overlap of potential target miRNAs of circUBE2Q2 predicted by miRanda and PITA. **B** Transfection efficiency of circUBE2Q2 probe in MKN-45 and BGC-823 cell lines. **C** Ten potential target miRNAs were pulled down and confirmed by qRT-PCR in MKN-45 cell line. **D** Dual-luciferase reporter assay used to detect the relative luciferase activity in MKN-45 cells cotransfected with luc-circUBE2Q2 and miR-370-3p mimics. **E** FISH for circUBE2Q2 and miR-370-3p in MKN-45 and BGC-823 cell lines, scale bar = 10 µm. **F** Expression level of miR-370-3p in GC tissues relative to matched normal tissues via qRT-PCR (*n* = 60). **G** CCK-8 analysis of the cell proliferation ability in MKN-45 cells cotransfected with circUBE2Q2-specific siRNA or miR-370-3p inhibitors. **H** Colony formation assay of the cell proliferation ability in cotransfected MKN-45 cells. **I** EdU analysis of the cell proliferation ability in treated MKN-45 cells. **J** Transwell invasion and migration assay in treated MKN-45 cells. **K** The growth of gastric cancer organoid models cotransfected with circUBE2Q2-specific siRNA or miR-370-3p inhibitors, scale bar = 25 µm. **L** Western blot analysis of EMT-related genes (TWIST, Snail, Slug, N-cadherin and E-cadherin) with proteins treated with circUBE2Q2-specific siRNA or miR-370-3p inhibitors in MKN-45 cell line. Data are expressed as the mean ± SD. *p < 0.05, **p < 0.01, ***p < 0.001.

Bioinformatic prediction databases including miRanda (http://www.miranda.org/) and PITA (https://genie.weizmann.ac.il/pubs/mir07/mir07_dyn_data.html) were used to cross-analyze the downstream miRNA targets of circUBE2Q2. Based on their conjugation scores, nine common miRNAs were selected to be the potential miRNAs absorbed by circUBE2Q2 in GC cells (Fig. 3A). We used a specific biotin-labeled circUBE2Q2 probe to absorb circUBE2Q2-associated miRNAs via a pull-down assay (Fig. 3B). Subsequently, we performed qRT-PCR assays to detect the expression levels of the nine predicted miRNAs in the sponge complex from circUBE2Q2 pull-down experiments in MKN-45 and BGC-823 cells. We only found the enrichment of circUBE2Q2 and miR-370-3p in both MKN-45 and BGC-823 cells compared with the interactions between the other miRNAs and circUBE2Q2 (Fig. 3C and Supplementary Fig. 1D). To further validate the direct binding between circUBE2Q2 and miR-370-3p, we performed a dual-luciferase reporter assay. Compared with the significant reduction in the luciferase signal caused by cotransfection of the wild-type circUBE2Q2 reporter genes and miR-370-3p mimics, no significant changes were observed after cotransfection of the mut3 (both) circUBE2Q2 reporter genes and miR-370-3p mimics (Fig. 3D and Supplementary Fig. 1E). The colocalization of circUBE2Q2 and miR-370-3p was confirmed in the cytoplasm of GC cells by FISH assay, which further suggested an interaction between circUBE2Q2 and miR-370-3p (Fig. 3E). A significantly upregulated expression of miR-370-3p in GC tissues was observed after performing qRT-PCR on 60 paired GC tumor tissues (Fig. 3F).

We used miR-370-3p inhibitors to examine whether the tumor promoter role of circUBE2Q2 knockdown could be rescued by miR-370-3p knockdown. We confirmed the transfection efficiency of miR-370-3p inhibitors via qRT-PCR (Supplementary Fig. 1F). We observed that the reduction in proliferation (Fig. 3G–I and Supplementary Fig. 2A–C) and metastasis (Fig. 3J and Supplementary Fig. 2D) caused by circUBE2Q2 knockdown was successfully reversed by miR-370-3p silencing. Moreover, the blocked growth of the GC organoid models by knocking down circUBE2Q2 was found to be significantly reversed by knocking down miR-370-3p and circUBE2Q2 at the same time (Fig. 3K). The effects of circUBE2Q2 knockdown on the protein levels of EMT-related factors were reversed by miR-370-3p inhibitors (Fig. 3L and Supplementary Fig. 2E).

These results proved that circUBE2Q2 could promote GC development by serving as a sponge of miR-370-3p.

### *STAT3* is a downstream target of miR-370-3p

**Fig. 4:**
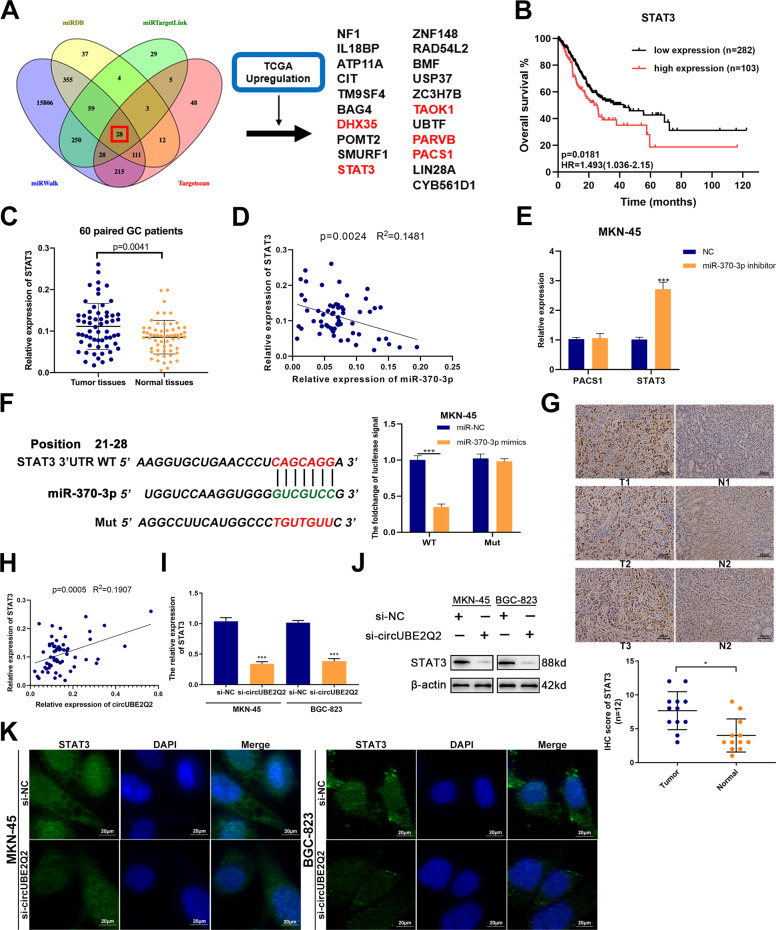
*STAT3* is a downstream target of miR-370-3p. **A** Venn diagram detailing the exploration of miR-370-3p downstream targets. **B** Overall survival analysis based on *STAT3* expression in TCGA GC patients. The optimal cutoff was calculated by X-tile software. **C** mRNA levels of *STAT3* in GC tissues and paired normal tissues detected by qRT-PCR (*n* = 60). **D** Correlation of *STAT3* and miR-370-3p expression in GC tissues and paired normal tissues (*n* = 60). **E** The expression of *PACS1* and *STAT3* after miR-370-3p silencing in MKN-45 cells measured by qRT-PCR. **F** A dual-luciferase reporter assay to determine the direct binding between miR-370-3p and *STAT3* in MKN-45 cells. **G** Expression of STAT3 detected via immunochemistry analysis (IHC) on 12 paired GC tumor tissues and normal stomach tissues, scale bar = 20 µm. **H** Correlation of *STAT3* and circUBE2Q2 expression in GC tissues (*n* = 60). **I** The expression of *STAT3* in GC cells transfected with circUBE2Q2-specific siRNA measured by qRT-PCR. **J** The protein levels of STAT3 in GC cells transfected with circUBE2Q2-specific siRNA measured by western blottings. **K** Immunofluorescence staining for STAT3 in treated MKN-45 and BGC-823 cells, scale bar = 20 µm. Data are expressed as the mean ± SD. *p < 0.05, **p < 0.01, ***p < 0.001.

To identify the target gene of miR-370-3p, four databases including miRDB (https://mirdb.org/), miRTargetLink (www.ccb-web.cs.uni-saarland.de/mirtargetlink/), miRWalk (https://www.ma.uni-heidelberg.de/apps/zmf/mirwalk/), and TargetScan (https://www.targetscan.org) were screened and 28 target genes were overlapped. In combination with the The Cancer Genome Atlas (TCGA) database, we screened 21 genes with upregulated expression (*p* < 0.05) from the 28 genes (Fig. 4A). Then, we performed survival analysis using the TCGA database and found that the high expression of four genes was significantly associated with shorter overall survival in GC patients, which were *STAT3*, *TAOK1*, *PARVB*, and *PACS1* (Fig. 4B and Supplementary Fig. 3A–C). We validated the significantly upregulated expression of *STAT3*, *PARVB*, and *PACS1* in 60 paired GC tissues and normal tissues (Fig. 4C and Supplementary Fig. 3D–F). Among them, the *PACS1* and *STAT3* expression levels exhibited a clear negative correlation with miR-370-3p, whereas we found no such correlation for *PARVB* (Fig. 4D and Supplementary Fig. 3G–H). qRT-PCR results indicated that *STAT3* expression was significantly upregulated in both MKN-45 and BGC-823 cells transfected with miR-370-3p inhibitors (Fig. 4E and Supplementary Fig. 3I). On the basis of the above results, we chose *STAT3* for further studies. Bioinformatics analysis were used to predict the binding sites between miR-370-3p and *STAT3*. Dual-luciferase reporter assays confirmed the direct interaction between miR-370-3p and *STAT3* in GC cells (Fig. 4F and Supplementary Fig. 3J). To further validate its expression pattern in GC tissues, we performed IHC on 12 of them (Fig. 4G). The IHC results also confirmed the significantly higher expression of *STAT3* in GC tissues than in adjacent normal tissues.

Moreover, a significant positive correlation between circUBE2Q2 and *STAT3* was found via qRT-PCR (Fig. 4H). Lower *STAT3* expression was observed via qRT-PCR and western blottings when we knocked down circUBE2Q2 in both MKN-45 and BGC-823 cells (Fig. 4I, J). We observed the same result when using an immunofluorescence (IF) assay, circUBE2Q2 knockdown inhibited the expression level of STAT3 in GC cells (Fig. 4K). In summary, as the target gene of miR-370-3p, *STAT3* may be a potential tumor promotor, which was regulated by circUBE2Q2.

### *STAT3* reverses the effects of circUBE2Q2 on GC

**Fig. 5:**
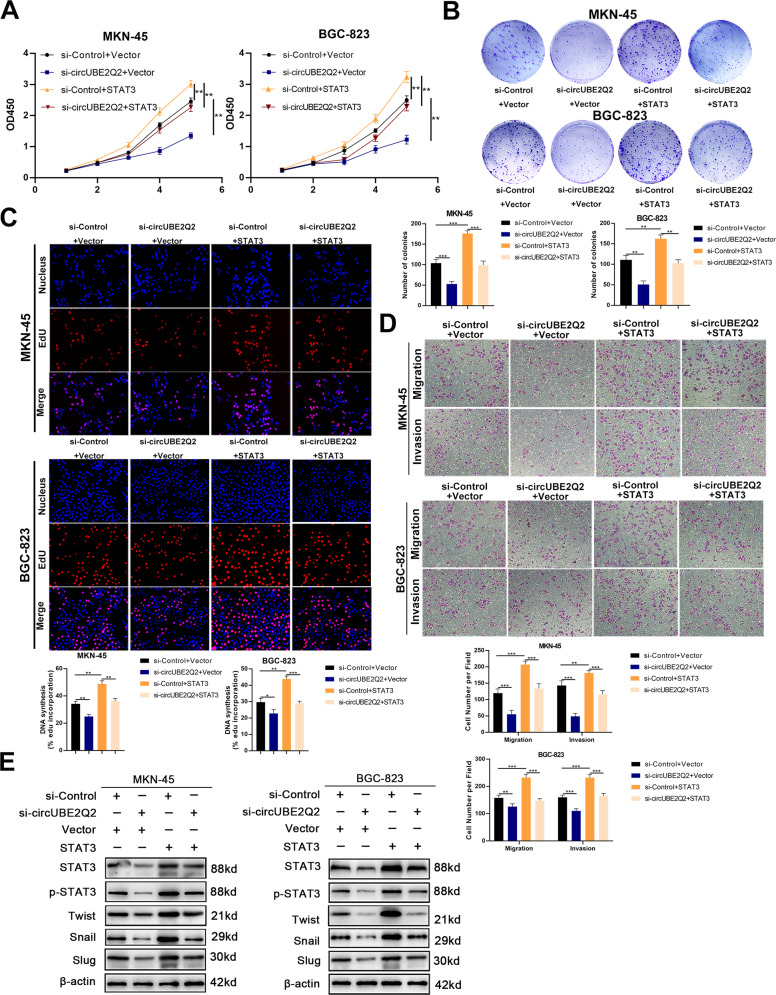
*STAT3* reverses the effects of circUBE2Q2 on GC. **A** CCK-8 analysis of the cell proliferation ability in MKN-45 and BGC-823 cell lines cotransfected with circUBE2Q2-specific siRNA or *STAT3* plasmids. **B** Colony formation assay of the cell proliferation ability in cotransfected MKN-45 and BGC-823 cell lines. **C** EdU analysis of the cell proliferation ability in treated MKN-45 and BGC-823 cell lines. **D** Transwell invasion and migration assay in treated MKN-45 and BGC-823 cell lines. **E** Western blot analysis of STAT3, p-STAT3, Twist, Snail, and Slug with proteins treated with circUBE2Q2-specific siRNA or *STAT3* plasmids in MKN-45 and BGC-823 cell lines. Data are expressed as the mean ± SD. *p < 0.05, **p < 0.01, ***p < 0.001.

We performed rescue experiments to verify whether circUBE2Q2 could mediate proliferation and metastasis of GC cells by regulating *STAT3*. The transfection efficiency of *STAT3* plasmids were confirmed via qRT-PCR (Supplementary Fig. 4A). The reduction of proliferation and metastasis of GC cells caused by circUBE2Q2 silencing was rescued by *STAT3* overexpression. (Fig. 5A–D).

*STAT3* has been reported to be constitutively activated (*p-STAT3*) in solid and hematological tumors [20, 33, 34]. Next, we detected the expression levels of STAT3, p-STAT3 (phospho Y705), and EMT-related factors in GC cells through western blottings. The effects of circUBE2Q2 knockdown on the protein levels of EMT-related factors were reversed by *STAT3* overexpression (Fig. 5E). Taken together, we demonstrated that, as a molecular sponge of miR-370-3p, circUBE2Q2 promotes GC progression through the *STAT3* signaling pathway.

### CircUBE2Q2/*STAT3* axis inhibits autophagy and promotes glycolysis

It has been reported that activated *STAT3/Bcl-2* pathway could inhibit autophagy, further promoting the proliferation of cancer cells [19, 21, 22]. Meanwhile, activated *STAT3* is also responsible for high metabolic activities to provide an environment for the survival of malignant tumors.

**Fig. 6:**
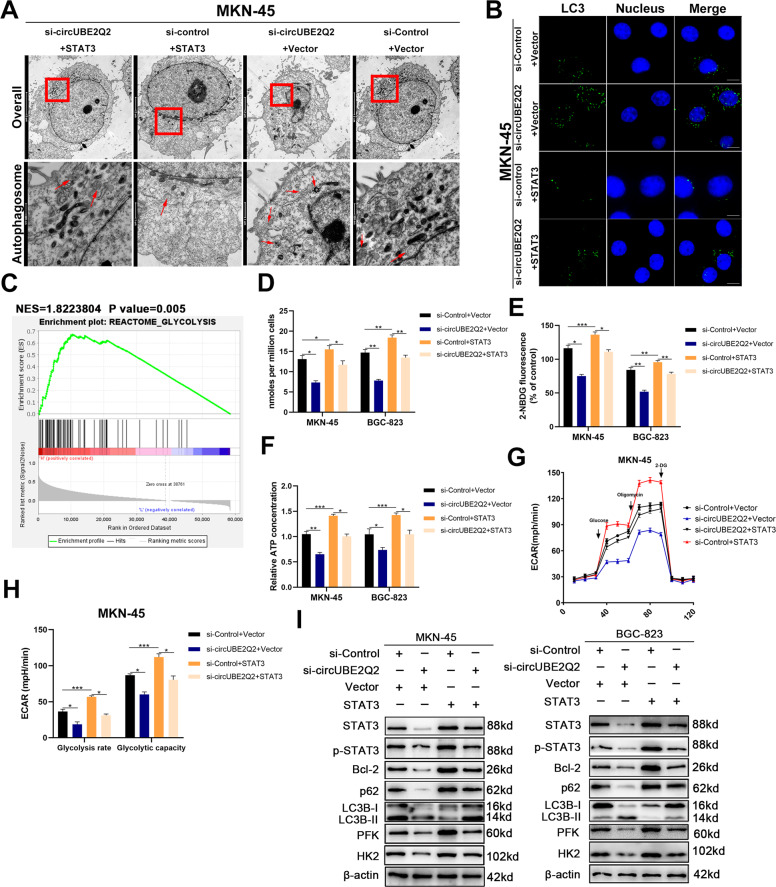
CircUBE2Q2 sponges miR-370-3p to inhibit autophagy and promote glycolysis. **A** The number of autophagosomes observed in MKN-45 cell lines cotransfected with circUBE2Q2-specific siRNA or *STAT3* plamids using transmission electron microscopy (TEM). **B** The role of circUBE2Q2/*STAT3* on autophagy using anti-LC3 via IF in MKN-45 cells, scale bar = 20 μm. **C** Gene set enrichment analysis (GSEA) identified a significant positive correlation between *STAT3* and the glycolysis pathway. **D**–**F** Lactate production, glucose uptake, and ATP production detected in GC cells. **G**, **H** Extracellular acidification rate (ECAR) measured in treated MKN-45 cells. **I** Protein levels of STAT3, p-STAT3, Bcl-2, p62, LC3B, PFK, and HK2 in treated MKN-45 and BGC-823 cell lines detected by western blottings. Data are expressed as the mean ± SD. *p < 0.05, **p < 0.01, ***p < 0.001.

To further verify whether circUBE2Q2 could inhibit serum deprivation-induced autophagy via *STAT3*, we used transmission electron microscopy (TEM) to observe the autophagosomes within MKN-45 and BGC-823 cells. More autophagosomes were observed in circUBE2Q2 knockdown GC cells. The number of autophagosomes was reversed in GC cells when circUBE2Q2 siRNA and *STAT3* plasmids were cotransfected at the same time (Fig. 6A and Supplementary Fig. 4B). Consistent with TEM results, the stronger green signal caused by circUBE2Q2 knockdown was reduced by *STAT3* plamids via IF (Fig. 6B and Supplementary Fig. 4C). Hence, we verified the inhibitory role of circUBE2Q2 on autophagy activation by regulating *STAT3*.

As the gene set enrichment analysis showed, *STAT3* was significantly positively correlated with glycolysis (Fig. 6C). To further validate the role of the circUBE2Q2/*STAT3* axis in glycolysis, we detected lactate production (Fig. 6D), glucose uptake (Fig. 6E), and ATP production (Fig. 6F). Cotransfection of *STAT3* plasmid and circUBE2Q2 siRNA at the same time could ameliorate the reduction in the aforementioned glycolysis activities caused by circUBE2Q2 knockdown alone. Next, the extracellular acidification rate was measured (Fig. 6G and Supplementary Fig. 3D). We observed that cirUBE2Q2 knockdown could reduce the glycolysis rate and glycolytic capacity, whereas circUBE2Q2 knockdown and *STAT3* overexpression could restore them (Fig. 6H and Supplementary Fig. 3E).

We detected the expression levels of STAT3, p-STAT3 (phospho Y705), Bcl-2, LC3, P62, and hexokinase-2 (HK2) and phosphofructokinase (PFK) in GC cells through western blottings. Knocking down circUBE2Q2 could promote the expression of autophagy marker LC3 and inhibit P62 and Bcl-2 expression, whereas further *STAT3* overexpression reversed these effects (Fig. 6I). Furthermore, simultaneous transfection of *STAT3* plasmid reversed the downregulated expression of glycolytic proteins, PFK and HK2, which was caused by circUBE2Q2 silencing (Fig. 6I).

In summary, the inhibition of autophagy and the promotion of glycolysis by the circUBE2Q2/miR-370-3p/*STAT3* axis were validated by the aforementioned results.

### Exosomal circUBE2Q2 regulates the *STAT3* signaling pathway and EMT in vitro, and promotes metastasis in vivo

**Fig. 7:**
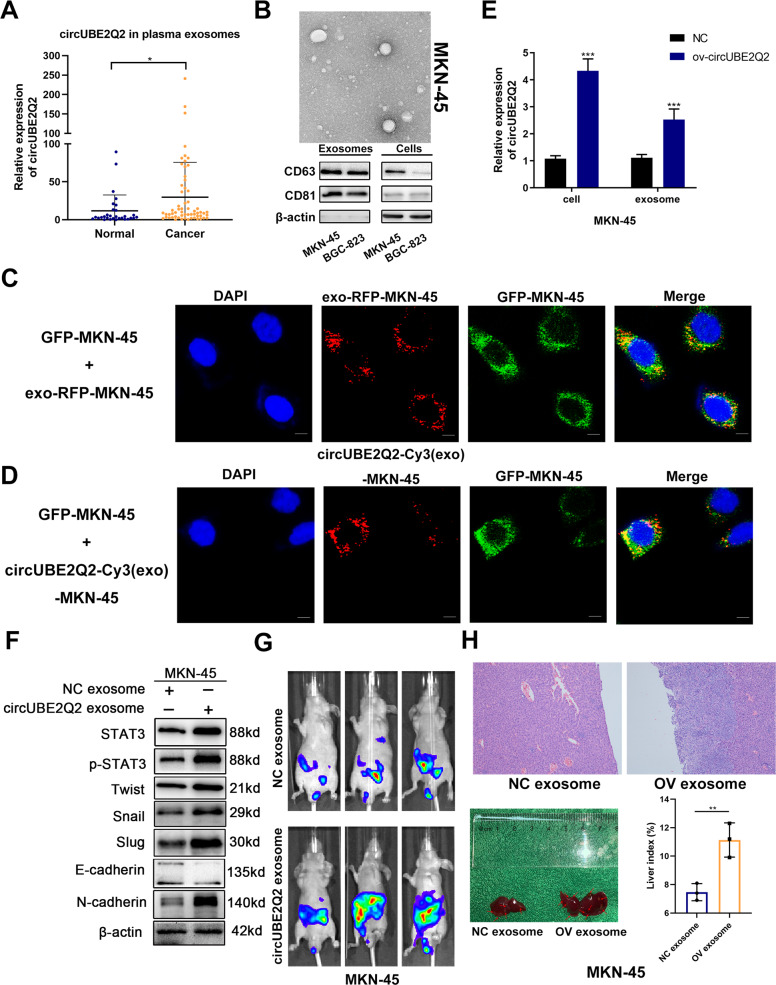
Exosomal circUBE2Q2 regulates the *STAT3* signaling pathway and EMT in vitro and promotes metastasis in vivo. **A** Expression level of circUBE2Q2 in the plasma exosomes from normal people (*n* = 30) and GC patients (*n* = 60) determined via qRT-PCR. **B** Existence and morphology of exosomes purified from MKN-45 cell medium (exosome-free FBS) determined by a transmission electron microscope (TEM) and exosomal markers CD63 and CD81 via western blottings. **C** The existence of the red exosome signals in the cytoplasm of MKN-45 cells, scale bar = 20 μm. **D** The red signals of circUBE2Q2 in the cytoplasm of GFP-labeled MKN-45 cells, scale bar = 20 μm. **E** Higher circUBE2Q2 expression in exosomes purified from cirUBE2Q2-overexpressing MKN-45 cells detected by qRT-PCR. **F** Protein levels of STAT3, p-STAT3, Twist, Snail, Slug, N-Cadherin, and E-Cadherin detected by western blottings. **G** Peritoneal metastasis in MKN-45 cells treated with NC or circUBE2Q2 OV exosomes measured by luciferase intensities. **H** The cancerous node size characterized by the liver tissues for H&E staining. Liver index = liver weight/body weight. Data are expressed as the mean ± SD. *p < 0.05, **p < 0.01, ***p < 0.001.

Exosomal communication has been recognized as an intracellular material transfer tool in recent years [35]. Thus, we determined whether exosomal communication exists between GC cells. We determined the expression level of circUBE2Q2 in plasma exosomes from 60 GC patients and 30 normal subjects. We found significantly higher circUBE2Q2 levels in the plasma exosomes of GC patients (Fig. 7A). Subsequently, the existence and morphology of exosomes purified from GC cell medium (exosome-free FBS) were determined by transmission electron microscopy (Fig. 7B and Supplementary Fig. 5A). The exosomal protein markers CD63 and CD81 within the exosome extraction were verified by western blottings, which indicated the existence of exosomes (Fig. 7B).

We established two cocultivation systems to trace exosomal communication between GC cells. In the first cocultivation system, we mixed the same amount of red fluorescent protein (RFP)-tagged CD63 plasmid-transfected GC cells (exo-RFP-MKN-45 and exo-RFP-BGC-823) with green fluorescent protein (GFP)-labeled GC cells for 72 h. We found the existence of red signals, which indicated exosomes in the cytoplasm of GFP-labeled tumor cells, suggesting the potential of exosomes to be a communication tool between GC cells (Fig. 7C and Supplementary Fig. 5B). In the second cocultivation system, we added exosomes purified from the Cy3-tagged circUBE2Q2-overexpressing plasmid GC cells into GFP-labeled GC cells. After 72 h, we observed red signals in the cytoplasm of GFP-labeled GC cells (Fig. 7D and Supplementary Fig. 5C). Meanwhile, we detected higher circUBE2Q2 expression in exosomes purified from circUBE2Q2-overexpressing GC cells (Fig. 7E and Supplementary Fig. 5D). Thus, we proved that circUBE2Q2 was present in exosomes released by GC cells.

As circUBE2Q2 has been proven to activate the *STAT3* pathway and promote EMT in GC cells. Meanwhile, exosomes have been reported to be biological vesicles that promote cancer metastasis via the “seed and soil” theory [27, 35]. Thus, we attempted to determine the biological function of exo-circUBE2Q2 in GC metastasis. We successfully detected the activated *STAT3* pathway and upregulated EMT markers in GC cells by coculturing them with exosomes from OV circUBE2Q2 GC cells (OV exosomes) for 72 h compared with coculturing with exosomes from control GC cells (NC exosomes) (Fig. 7F and Supplementary Fig. 5E). Next, we detected the role of exo-circUBE2Q2 in metastasis in vivo. We injected GC cells into the hepatic portal vein and abdominal cavity of BALB/c nude mice, respectively, and then cocultured them with OV exosomes and NC exosomes. We observed increased peritoneal metastasis in the mice, under OV exosome treatments after 4 weeks (Fig. 7G and Supplementary Fig. 5F). Moreover, we observed more metastatic nodes in the livers of OV exosome-treated mice and collected the liver tissues for hematoxylin and eosin staining (Fig. 7H and Supplementary Fig. 5G) to validate the cancerous node size, which was consistent with the luciferase intensity.

In summary, the role of circUBE2Q2 in promoting metastasis through exosomal communication was confirmed.

### Combination of circUBE2Q2 knockdown and stattic has a stronger tumor suppressor role

**Fig. 8:**
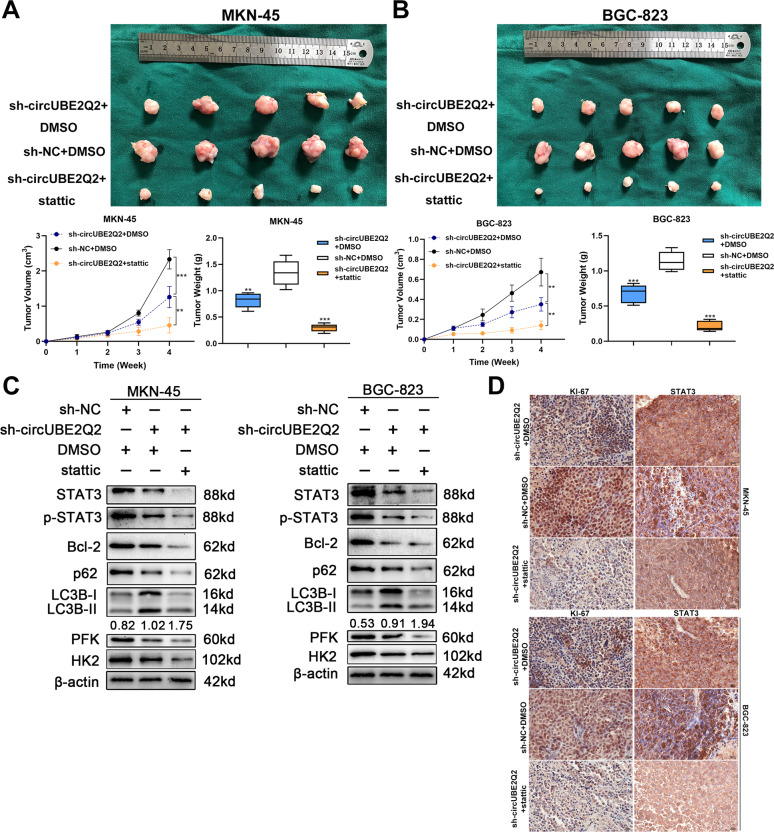
Combination of circUBE2Q2 knockdown and stattic has a stronger tumor suppressor role. **A**, **B** Tumor volume and weight in mice that received subcutaneous injections. **C** Protein levels of STAT3, p-STAT3, Bcl-2, p62, LC3B, PFK, and HK2 in xenograft tumor tissues detected by western blottings. **D** Immunohistochemistry staining (IHC) of STAT3 and Ki-67 in respective xenograft tumor tissues. Data are expressed as the mean ± SD. *p < 0.05, **p < 0.01, ***p < 0.001.

We proved that knocking down circUBE2Q2 could suppress the malignancy of GC cells in vitro by blocking the *STAT3*-mediated oncogenic pathway. Thus, we investigated the tumor suppressor role of knocking down circUBE2Q2 in vivo and whether combination of circUBE2Q2 knocking down and stattic (a common *STAT3* inhibitor) could result in additive or synergistic effects on the growth of GC in vivo. We observed that stattic reduced the growth of the xenograft tumors in nude mice, and that circUBE2Q2 short hairpin RNA (shRNA) together with stattic had a better therapeutic effect on limiting tumor growth (Fig. 8A, B). Subsequently, we found that combination treatment had a better effect on reducing the phosphorylation of the *STAT3/Bcl-2* pathway via western blottings on xenograft tumor tissues (Fig. 8C). In addition, combination treatment could reduce glycolysis and promote autophagy in vivo more significantly compared with the control group (Fig. 8C).

In addition, we observed the lower expression of STAT3 and Ki-67 in tumor tissues in the stattic treatment group, and the much lower expression levels in the circUBE2Q2 shRNA combination group than in the negative control mouse tumor tissues via IHC (Fig. 8D).

In conclusion, we found that combination treatment of stattic and circUBE2Q2 silencing has a stronger synergistic effect on tumor suppression in vivo through the *STAT3* signaling pathway.

## Discussion

circRNAs have been confirmed to play regulatory roles in various cancers, including breast cancer, colorectal cancer, hepatocellular carcinoma, and bladder cancer [8–10, 36]. Emerging evidence proves that circRNAs could play functional roles as tumor regulators by acting as miRNA sponges, transcription factors, or RBPs [5, 12–14]. In our study, we confirmed that circUBE2Q2 could serve as a sponge of miR-370-3p to regulate the malignant progression of GC. Although miR-370-3p has been reported to regulate paclitaxel resistance of ovarian cancer, promote the proliferation of glioma, regulate breast cancer metastasis, etc., the role of miR-370-3p in GC remains unclear [37–39].

We next confirmed *STAT3* as a competing endogenous RNA of circUBE2Q2, and that the circUBE2Q2/miR-370-3p axis could promote GC malignant progression. Subsequently, we confirmed that circUBE2Q2/*STAT3* axis could block autophagy and promote glycolysis, favoring GC growth. Although our research has proven that the circUBE2Q2/miR-370-3p/*STAT3* axis mediates autophagy impairment, and that high-level glycolysis can promote the development of GC, whether there is mutual regulation between autophagy and glycolysis, and whether the regulatory relationship between them has a positive feedback regulating effect on GC still need more in-depth research.

Exosomes are a subgroup of extracellular vesicles, which can transmit cellular molecular constituents including miRNAs, circRNAs, and lncRNAs, to promote intercellular communication and further affect the development of cancer [40, 41]. It has been reported that exosomes could promote the progression of GC through autocrine in 2009 [42]. Xie et al. [43] also confirmed exosomes with upregulated circSHKBP1 promoted the growth of cocultured GC cells. We established two cocultivation systems and confirmed that circUBE2Q2 was present in exosomes released by GC cells. We found that exosomal circUBE2Q2 could regulate the *STAT3* signaling pathway and EMT in vitro, and promotes metastasis in vivo. Thus, these results indicated that with the overexpression of circUE2Q2 in GC cells, more circUBE2Q2 can be loaded into exosomes, thus affecting the biological functions of GC cells and the activation of downstream pathways in an autocrine or paracrine manner. However, the in-depth mechanism of exosomal circUBE2Q2 promoting the malignant progression of GC remains unclear and still needs further research.

Further in vivo experiments confirmed that the combination of circUBE2Q2 knockdown and *STAT3* inhibitors inhibited tumor growth more significantly than knocking down circUBE2Q2 alone. Targeting the circUBE2Q2/*STAT3* axis is expected to provide new ideas for targeted therapy of GC.

In summary, we utilized several in vivo and in vitro experiments, and confirmed that circUBE2Q2 could promote GC cell proliferation and migration by sponging miR-370-3p and activating the *STAT3* pathway. These findings suggest that circUBE2Q2 could be a key tumor promoter and a potential therapeutic target for GC.

## Supplementary information

Supplementray figure legends

Supplementary figure 1

Supplementary figure 2

Supplementary figure 3

Supplementary figure 4

Supplementary figure 5

TABLE S1 S2

## Data Availability

All data generated or analyzed during this study are included in this published article and its Additional Files.
