# Supplementary material for: Circular RNA UBE2Q2 promotes malignant progression of gastric cancer by regulating signal transducer and activator of transcription 3-mediated autophagy and glycolysis
Source: Cell Death Dis. 2021 Oct 5;12(10):910. doi: 10.1038/s41419-021-04216-3 (PMC8492724; doi:10.1038/s41419-021-04216-3)
Supplement: Supplementary file 1 — Supplementray figure legends [file 41419_2021_4216_MOESM1_ESM.docx]

**Supplementary Figure 1.** (A). Clustered heatmap showing the expression level of the top 10 circRNAs in GC cell lines. (B). The volcano plots illustrated the expression variations of these circRNAs in cancerous tissues compared to matched noncancerous tissues. (C). Transfection efficiency of cricUBE2Q2 overexpression plasmid and siRNAs detected by qRT-PCR. (D). Ten potential target miRNAs were pulled down and confirmed by qRT-PCR in BGC-823 cell line.

(E). Dual luciferase reporter assay used to detect the relative luciferase activity in BGC-823 cells co-transfected with luc-circUBE2Q2 and miR-370-3p mimics. (F). Transfection efficiency of miR-370-3p mimics and inhibitors detected by qRT-PCR. Data are expressed as the mean ± SD. *p < 0.05, **p < 0.01, ***p < 0.001.

**Supplementary Figure 2.** (A). CCK8 analysis of the cell proliferation ability in BGC-823 cells cotransfected with circUBE2Q2-specific siRNA or miR-370-3p inhibitors. (B). Colony formation assay of the cell proliferation ability in cotransfected BGC-823 cells. (C). EdU analysis of the cell proliferation ability in treated BGC-823 cells. (D). Transwell invasion and migration assay in treated BGC-823 cells. (E). Western blot analysis of EMT-related genes (TWIST, snail, slug, N-cadherin and E-cadherin) with proteins treated with circUBE2Q2-specific siRNA or miR-370-3p inhibitors in BGC-823 cell line. Data are expressed as the mean ± SD. *p < 0.05, **p < 0.01, ***p < 0.001.

**Supplementary Figure 3.** (A-C). Overall survival analysis based on TAOK1, PARVB and PACS1 expression in TCGA GC patients. The optimal cut-off was calculated by X-tile software. (D-F). mRNA levels of TAOK1, PARVB and PACS1 in GC tissues and paired normal tissues detected by qRT-PCR. (G). Correlation of PACS1 and miR-370-3p expression in GC tissues and paired normal tissues (n=60). (H). Correlation of PARVB and miR-370-3p expression in GC tissues and paired normal tissues (n=60). (I). The expression of PACS1 and STAT3 after miR-370-3p silencing in BGC-823 cells measured by qRT-PCR. (J). A dual-luciferase reporter assay to determine the direct binding between miR-370-3p and STAT3 in MKN-45 cells. Data are expressed as the mean ± SD. *p < 0.05, **p < 0.01, ***p < 0.001.

**Supplementary Figure 4.** (A). Transfection efficiency of STAT3 plasmids in MKN-45 and BGC-823 cell lines detected by qRT-PCR. (B). The number of autophagosomes observed in BGC-823 cell lines cotransfected with circUBE2Q2-specific siRNA or STAT3 plamids using transmission electron microscopy (TEM). (C). The role of circUBE2Q2/STAT3 on autophagy using anti-LC3 via IF in MKN-45 cells, scale bar = 20 μm. (D, E). Extracellular acidification rate (ECAR) measured in treated BGC-823 cells. Data are expressed as the mean ± SD. *p < 0.05, **p < 0.01, ***p < 0.001.

**Supplementary Figure 5.** (A). Existence and morphology of exosomes purified from MKN-45 cell medium (exosome-free FBS) determined by a transmission electron microscope (TEM). (B). The existence of the red exosome signals in the cytoplasm of BGC-823 cells, scale bar = 20 μm. (C). The red signals of circUBE2Q2 in the cytoplasm of GFP-labelled BGC-823 cells, scale bar = 20 μm. (D). Higher circUBE2Q2 expression in exosomes purified from cirUBE2Q2-overexpressing BGC-823 cells detected by qRT-PCR. (E). Protein levels of STAT3, p-STAT3, Twist, Snail, Slug, N-Cadherin and E-Cadherin detected by western-blots. (F). Peritoneal metastasis in BGC-823 cells treated with NC or circUBE2Q2 OV exosomes measured by luciferase intensities. (G). The cancerous node size characterized by the H&E staining. Liver index=liver weight/ body weight. Data are expressed as the mean ± SD. *p < 0.05, **p < 0.01, ***p < 0.001.
