# Supplementary figures and images for: Circular RNA UBE2Q2 promotes malignant progression of gastric cancer by regulating signal transducer and activator of transcription 3-mediated autophagy and glycolysis
Source: Cell Death Dis. 2021 Oct 5;12(10):910. doi: 10.1038/s41419-021-04216-3 (PMC8492724; doi:10.1038/s41419-021-04216-3)

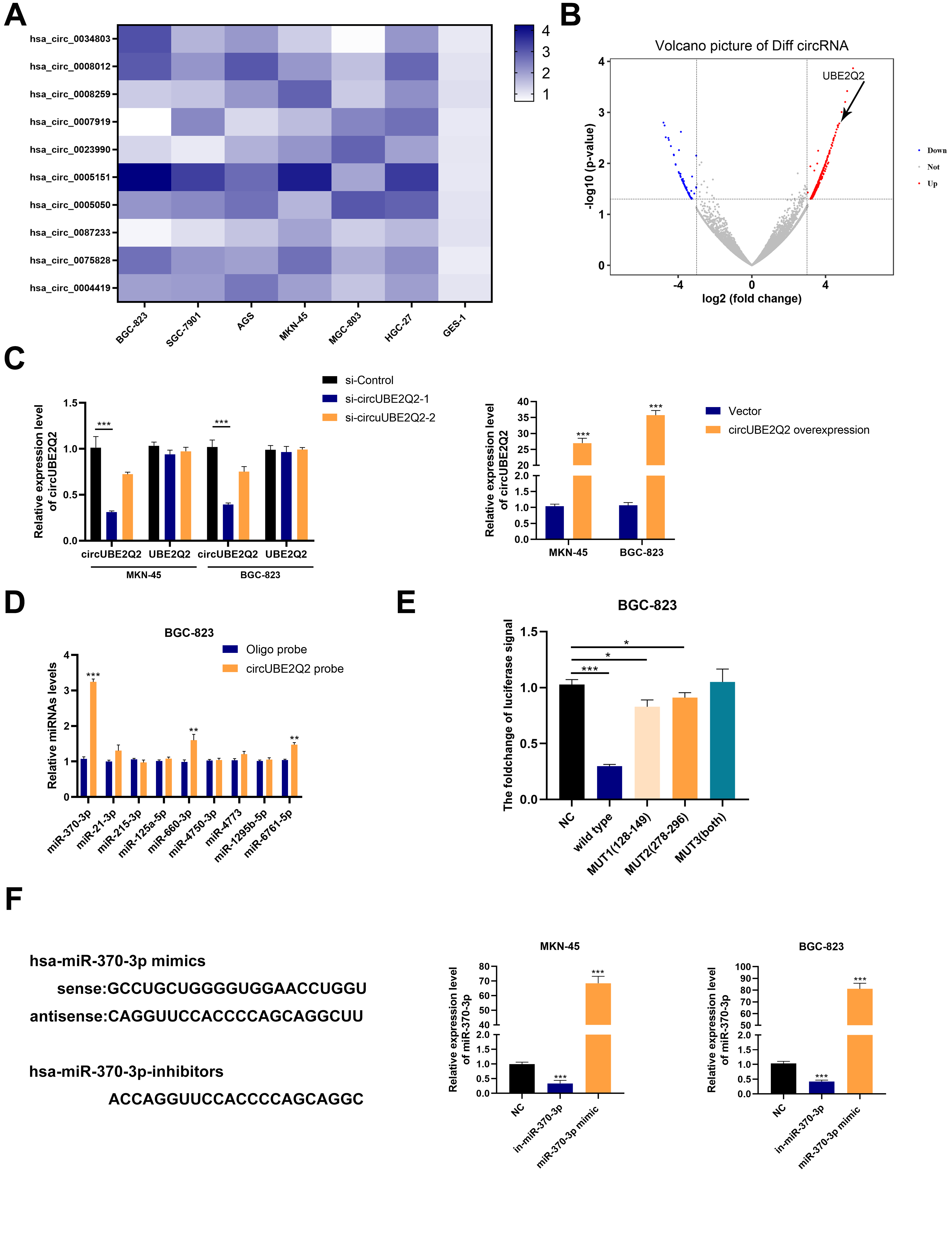

Supplement: Supplementary file 2 — Supplementary figure 1 [file 41419_2021_4216_MOESM2_ESM.png]

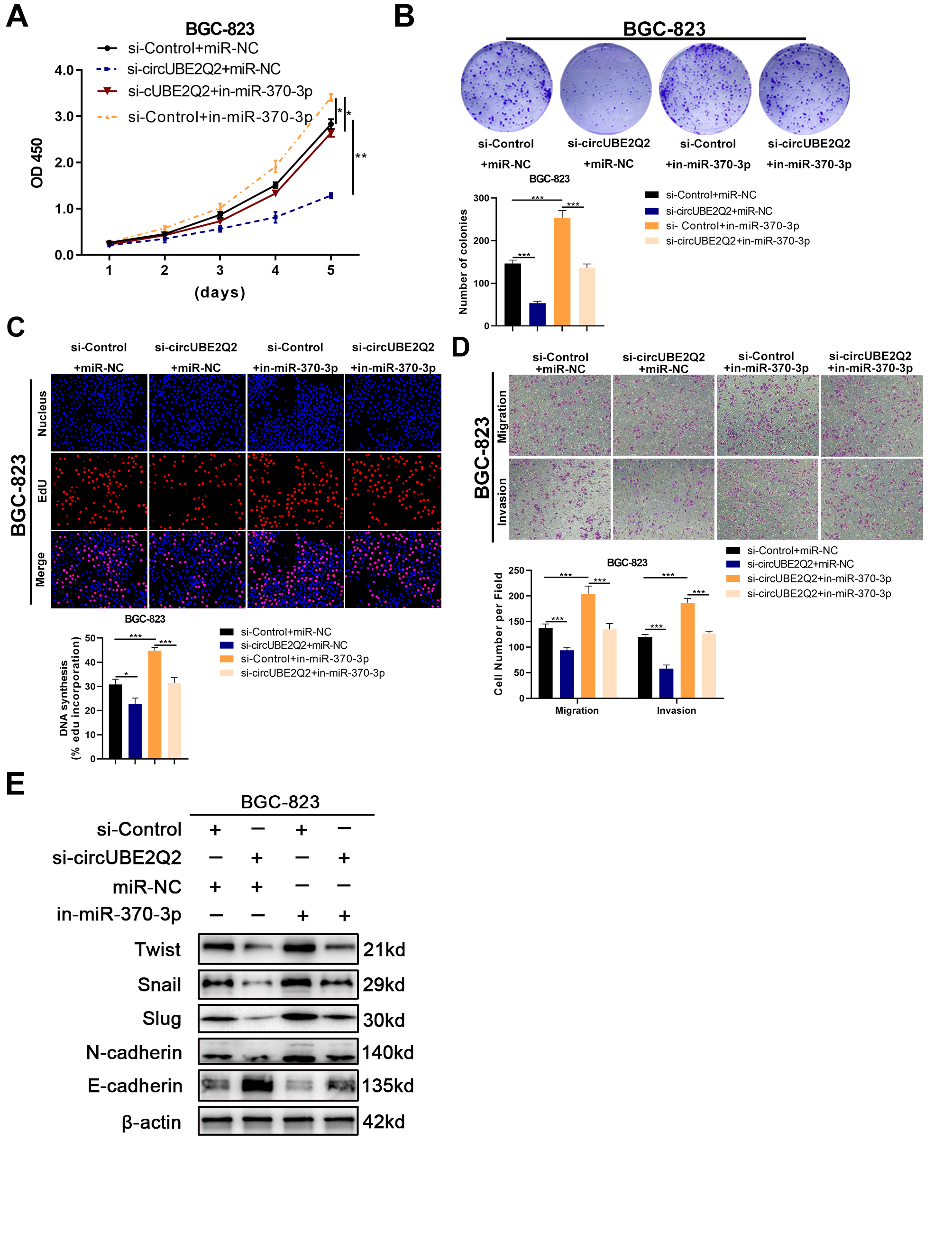

Supplement: Supplementary file 3 — Supplementary figure 2 [file 41419_2021_4216_MOESM3_ESM.png]

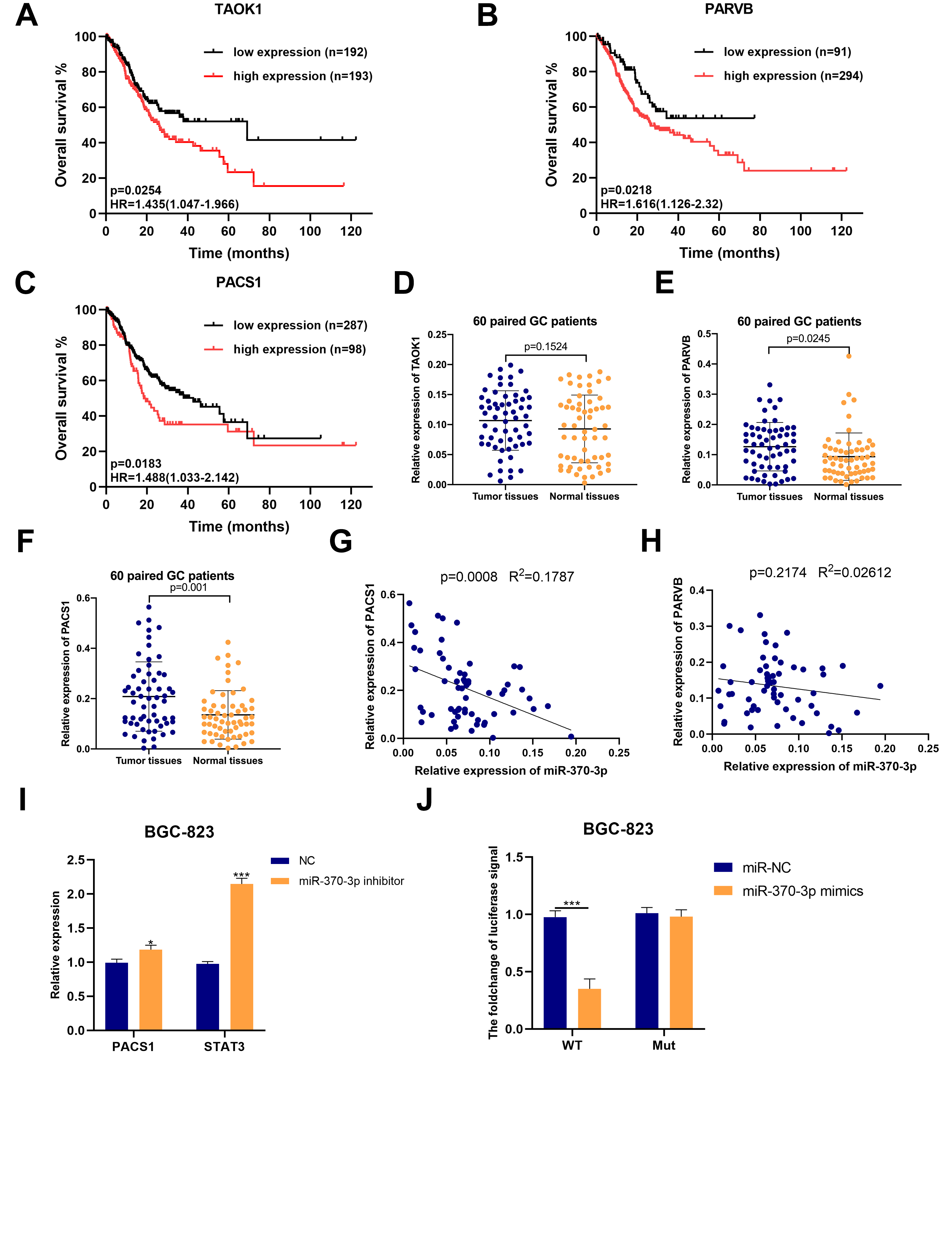

Supplement: Supplementary file 4 — Supplementary figure 3 [file 41419_2021_4216_MOESM4_ESM.png]

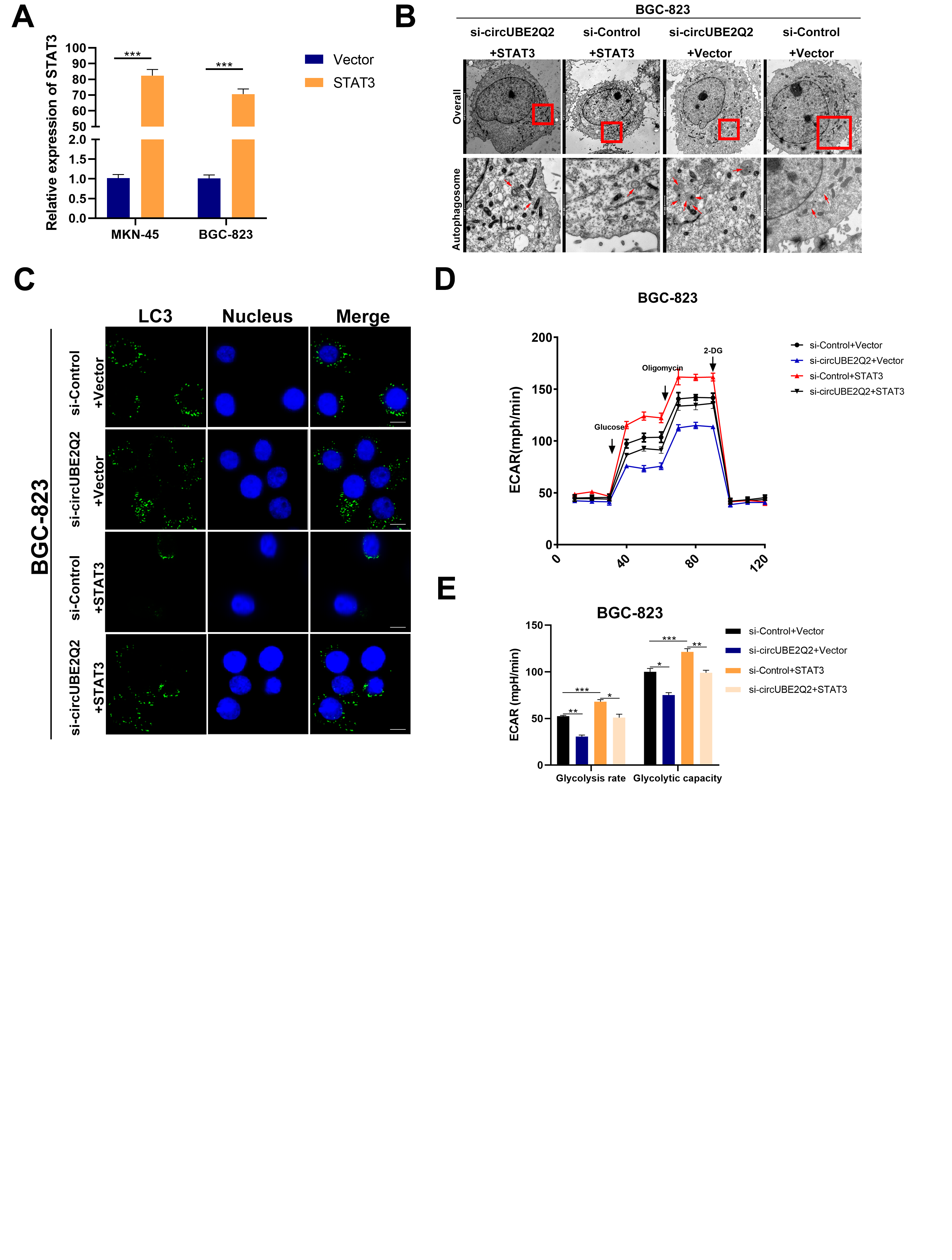

Supplement: Supplementary file 5 — Supplementary figure 4 [file 41419_2021_4216_MOESM5_ESM.png]

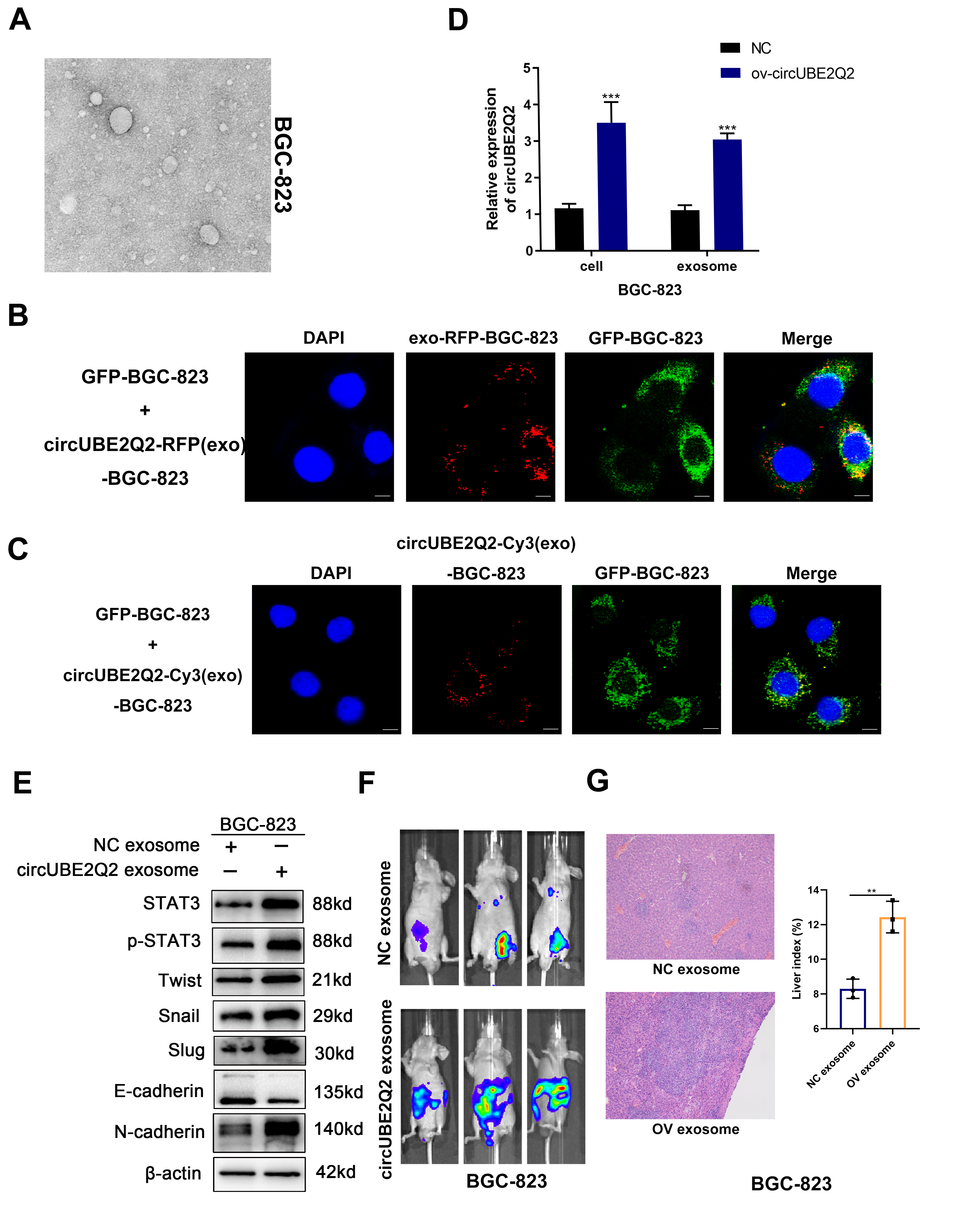

Supplement: Supplementary file 6 — Supplementary figure 5 [file 41419_2021_4216_MOESM6_ESM.png]
