# Supplementary material for: Circular RNA UBE2Q2 promotes malignant progression of gastric cancer by regulating signal transducer and activator of transcription 3-mediated autophagy and glycolysis
Source: Cell Death Dis. 2021 Oct 5;12(10):910. doi: 10.1038/s41419-021-04216-3 (PMC8492724; doi:10.1038/s41419-021-04216-3)
Supplement: Supplementary file 7 — TABLE S1 S2 [file 41419_2021_4216_MOESM7_ESM.docx]

Supplementary Table 1

| Primer sequence |  |
| --- | --- |
| circTMEM87A | Forward: 5’-CAATCAGCGCTTTTCAGATACC-3’ Reverse: 5’-ACAGGAGGCTAAGGAGAATGGA-3’ |
| circNFKB1 | Forward: 5’-CTCAAACTTAATTGGCT-3’ Reverse: 5’-CTTTTCACTAGAGGCAC-3’ |
| circLMO7 | Forward: 5’-CAAATCGAGTCACTGTCAAGCA-3’ Reverse: 5’-TCACACAGCAGAACACCATTTT-3’ |
| circABR | Forward: 5’-TCGTGGACAAGATCATGGGC-3’ Reverse: 5’-CCAAGAACCCCGAGAGAACC-3’ |
| circNOX4 | Forward: 5’-ACAACTGTTCCTGGCCTGAC-3’ Reverse: 5’-GGATAAGGCTGCAGTTGAGG-3’ |
| circUBE2Q2 | Forward: 5’-CAGAAGGTTGACCCTGATAGTCC-3’ Reverse: 5’-GCTTGCACTGACCCAGACACT-3’ |
| circXPO1 | Forward: 5’-AGGAACCAGTGCGAAGCAAA-3’ Reverse: 5’-AAATTGTGTCGACTCTTGTCCA-3’ |
| circPCSK5 | Forward: 5’-AGAGTGTTCATCCACGCTGG-3’ Reverse: 5’-GCGAGTCCCATGCGATTTTC-3’ |
| circCASC15 | Forward: 5’-GGAAGTGGCTAATGGATCTG-3’ Reverse: 5’-ATGGAGAACAGCCATCCATG-3’ |
| circR3HCC1L | Forward: 5’-CTATTGAAGCCTGCACCTGC-3’ Reverse: 5’-TTCGGAGAACCAAACCCAGG-3’ |
| linear UBE2Q2 | Forward: 5’-ACTGCAGAAGGTTGACCCTG-3’ Reverse: 5’-ACCACTCGAACAAATGGAGGA-3’ |
| miR-21-3p | Forward: 5'-CAACACCAGTCGATGGGCTGT-3' Reverse: General downstream primer  5'-CAGTGCGTGTCGTGGAGT-3' |
| miR-370-3p | Forward: 5'-GCCTGCTGGGGTGGAACCTGGT-3' Reverse: General downstream primer' |
| miR-215-3p | Forward: 5'-TCTGTCATTTCTTTAGGCCAATA-3' Reverse: General downstream primer' |
| miR-125a-5p | Forward: 5'-TCCCTGAGACCCTTTAACCTGTGA-3' Reverse: General downstream primer' |
| miR-660-3p | Forward: 5'-ACCTCCTGTGTGCATGGATTA-3' Reverse: General downstream primer' |
| miR-4750-3p | Forward: 5'-CCTGACCCACCCCCTCCCGCAG-3' Reverse: General downstream primer |
| miR-4773 | Forward: 5'-CAGAACAGGAGCATAGAAAGGC-3' Reverse: General downstream primer' |
| miR-1295b-5p | Forward: 5'-CACCCAGATCTGCGGCTAAT-3' Reverse: General downstream primer' |
| miR-6761-5p | Forward: 5'-TCTGAGAGAGCTCGATGGCAG-3' Reverse: General downstream primer' |
| ­STAT3 | Forward: 5'-CTCTGCCGGAGAAACAGGTG-3' Reverse: 5'-GCGTCTCTTCATCTCTCCCG-3' |
| PACS1 | Forward: 5'-GCGACTTTCTGTGAGAATGAGA -3' Reverse: 5'-ACAGCATCACTGTGGCCTAA-3' |
| β-actin | Forward: 5'-ATTGCCGACAGGATGCAGAA-3' Reverse: 5'-GCTGATCCACATCTGCTGGAA-3' |
| U6 | Forward: 5'-CTCGCTTCGGCAGCACA-3' Reverse: General downstream primer’ |
| Transfection sequence |  |
| si-circUBE2Q2-1 | Sense: 5’-GGAGGGGUGCAGUGUCUGGTT-3’  Anti-sense: 5’-CCAGACACUGCACCCCUCCTT-3’ |
| si-circUBE2Q2-2 | Sense: 5’-GUUCUCUCAGGAGGGGUGCTT-3’  Anti-sense: 5’-GCACCCCUCCUGAGAGAACTT-3’ |
| miR-370-3p mimics | Sense: 5’-GCCUGCUGGGGUGGAACCUGGU-3’  Anti-sense: 5’-CAGGUUCCACCCCAGCAGGCUU-3’ |
| miR-370-3p inhibitors | Sense: 5’-ACCAGGUUCCACCCCAGCAGGC-3’ |
| Fluorescent probe sequence |  |
| circUBE2Q2 | 5'-Fam-ACTGACCCAGACACTGCACC  CCTCCTGAGAGAACAGGTAA-Fam-3' |
| miR-370-3p | 5'-Cy3-ACCAGGTTCCACCCCAGCAGGC-Cy3–3' |
| Luciferase reporter gene mutation sequence |  |
| circUBE2Q2(mut128-149) | 5'-CCGUACAAUUUGACGUCUUCCA-3' |
| circUBE2Q2(mut278-296) | 5'-AUGGACAAGAGAGUCCUCC-3' |

Supplementary Table 2

| Primary antibody |  |  |
| --- | --- | --- |
| Twist | Cell signaling Technology | #69366 |
| Snail | Cell signaling Technology | #3879 |
| Slug | Cell signaling Technology | #9585 |
| STAT3 | proteintech | 10253-2-AP |
| N-cadherin | Cell signaling Technology | #13116 |
| E-cadherin | Cell signaling Technology | #14472 |
| Bcl-2 | proteintech | 12789-1-AP |
| p-STAT3(Y702) | abcam | ab267373 |
| HK2 | proteintech | 22029-1-AP |
| p62 | proteintech | 18420-1-AP |
| LC3B | Cell signaling Technology | #3868 |
| PFK | Abcam | ab181861 |
| CD63 | proteintech | 25682-1-AP |
| CD81 | proteintech | 27855-1-AP |
| β-actin | Cell signaling Technology | #4970 |
| Secondary antibody |  |  |
| Anti-rabbit IgG | Cell signaling Technology | #7074 |
| Anti-mouse IgG | Cell signaling Technology | #7076 |
